# Hydrostatic Compress Force Enhances the Viability and Decreases the Apoptosis of Condylar Chondrocytes through Integrin-FAK-ERK/PI3K Pathway

**DOI:** 10.3390/ijms17111847

**Published:** 2016-11-07

**Authors:** Dandan Ma, Xiaoxing Kou, Jing Jin, Taotao Xu, Mengjie Wu, Liquan Deng, Lusi Fu, Yi Liu, Gang Wu, Haiping Lu

**Affiliations:** 1 School of Stomatology, Zhejiang Chinese Medical University, Hangzhou 310053, China; whiterose1989@126.com (D.M.); jinjingtoday@163.com (J.J.); totti.12345@163.com (L.D.); fulusi@126.com (L.F.); 2 Department of Oral Implantology and Prosthetic Dentistry, Academic Centre for Dentistry Amsterdam (ACTA), VU University Amsterdam and University of Amsterdam, MOVE Research Institute, Gustav Mahlerlaan 3004, Amsterdam 1081LA, Nord-Holland, The Netherlands; liuzzd@163.com; 3 Department of Orthodontics, Peking University School and Hospital of Stomatology, Beijing 100081, China; kouxiaoxing@gmail.com; 4 Department of Orthopaedic Surgery, the First Affiliated Hospital of Zhejiang Chinese Medical University, Hangzhou 310006, China; nacle1990829@163.com; 5 Department of Orthodontics, Stomatology Hospital Affiliated to Zhejiang University, Hangzhou 310053, China; baobalala@sina.cn

**Keywords:** integrin, FAK, ERK, PI3K, mandibular condyle, chondrocyte, apoptosis

## Abstract

Reduced mechanical stimuli in many pathological cases, such as hemimastication and limited masticatory movements, can significantly affect the metabolic activity of mandibular condylar chondrocytes and the growth of mandibles. However, the molecular mechanisms for these phenomena remain unclear. In this study, we hypothesized that integrin-focal adhesion kinase (FAK)-ERK (extracellular signal–regulated kinase)/PI3K (phosphatidylinositol-3-kinase) signaling pathway mediated the cellular response of condylar chondrocytes to mechanical loading. Primary condylar chondrocytes were exposed to hydrostatic compressive forces (HCFs) of different magnitudes (0, 50, 100, 150, 200, and 250 kPa) for 2 h. We measured the viability, morphology, and apoptosis of the chondrocytes with different treatments as well as the gene, protein expression, and phosphorylation of mechanosensitivity-related molecules, such as integrin α2, integrin α5, integrin β1, FAK, ERK, and PI3K. HCFs could significantly increase the viability and surface area of condylar chondrocytes and decrease their apoptosis in a dose-dependent manner. HCF of 250 kPa resulted in a 1.51 ± 0.02-fold increase of cell viability and reduced the ratio of apoptotic cells from 18.10% ± 0.56% to 7.30% ± 1.43%. HCFs could significantly enhance the mRNA and protein expression of integrin α2, integrin α5, and integrin β1 in a dose-dependent manner, but not ERK1, ERK2, or PI3K. Instead, HCF could significantly increase phosphorylation levels of FAK, ERK1/2, and PI3K in a dose-dependent manner. Cilengitide, the potent integrin inhibitor, could dose-dependently block such effects of HCFs. HCFs enhances the viability and decreases the apoptosis of condylar chondrocytes through the integrin-FAK-ERK/PI3K pathway.

## 1. Introduction

The temporomandibular joint (TMJ) is an important contributor to the growth of the mandible length and height. Mandibular condylar cartilage in the temporomandibular joint is the greatest growth center in the craniofacial complex. The proper proliferation, maturation, and endochondral ossification of mandibular condylar cartilages are of paramount importance for the maxillofacial skeleton morphogenesis and temporomandibular joint functions [1].

Sufficient mechanical stimulus during the mandible movement plays a critical role in maintaining the survival, proper growth, and functions of mandibular condylar cartilage. Reduced mechanical stimuli in many pathological cases—such as soft diet, hemimastication, and limited masticatory movements—can significantly affect the metabolic activity of mandibular condylar cartilages and the growth of mandible. In clinical cases, such compromised mechanical stimuli could lead to significantly less growth activity of the condylar cartilage and macroscopic growth of the lower jaw [2,3,4]. In simulated animal models, reduced masticatory forces could result in a lower number of chondrocytes and cell layers, thinner condylar cartilages, and a reduction in trabecular bone density deep to the cartilage [5,6,7,8]. The cellular mechanisms accounting for these pathological changes may be the marked reduction in metabolic activities, such as proliferative activity and proteoglycan synthesis in mandibular condylar cartilage [9]. Besides, the inhibited growth of the developing mandible was also found to be related to the increased apoptosis and death of chondrocytes at the proliferation stage of the reserve zone [10,11]. These results indicated that sufficient loading was important for preventing apoptosis and maintaining the metabolic activities of condylar chondrocytes [9]. However, the molecular signaling transduction pathway accounting for the effects of extracellular mechanical forces on intracellular metabolic activities of mandibular condylar chondrocytes remains largely unveiled.

Integrins, a group of heterodimeric cell-surface molecules, are one of the most important mediators for the extracellular-intracellular signaling transduction. Each integrin molecule consists of two different chains: α and β subunits. In mammals, there are 18 α and 8 β subunits [12]. Integrin α5β1, the classic receptor for fibronectin, seems to be very important for the transmission of mechanical forces from extracellular matrix to cells [13,14]. Following the activation of integrins, focal adhesion kinase (FAK), as one of the main integrin-associated signaling and adaptor molecules, will be phosphorylated to mediate the intracellular effect of mechanical forces [15,16,17]. The involvement of integrin α5β1-FAK signaling pathways in mediating the biological response to mechanical stimuli was further corroborated by the in vivo finding that an elevated mechanical stimulus resulted in a significantly higher level of the integrin α5β1 and FAK than the sides with a reduced mechanical stimulus [18]. In addition, integrin α2 was shown to be also associated with the survival of condylar cartilage [19]. FAK can further cooperatively interact with receptor tyrosine kinase signaling to regulate adhesion, migration, survival, proliferation, polarization, and differentiation [20]. For example, FAK acted as an upstream regulator of the ERK1/2 (extracellular signal–regulated kinase) signaling upon mechanical stimulation in many cell types [21,22]. Following activation, phosphorylated ERK1/2 could translocate to the nucleus and activate transcription factors that are involved in response to mechanical stimulation [23]. FAK could also modulate cell apoptosis by involving PI3K (phosphatidylinositol-3-kinase) pathways [24].

Hitherto, it remains unclear whether integrin-FAK-ERK/PI3K signaling pathways mediate the cellular response of condylar chondrocytes to mechanical loading. In this study, we adopted an apparatus for administrating hydrostatic compressive forces (HCFs) to mimic the mechanical microenvironment of the masticatory forces. Thereby, we wished to approach the mechanotransduction mechanisms of primary condylar chondrocytes by assessing their proliferation, apoptosis, and signaling pathways under the stimulation of different-magnitude HCFs.

## 2. Results

### 2.1. Characterization of Primary Condylar Chondrocytes

**Figure 1:**
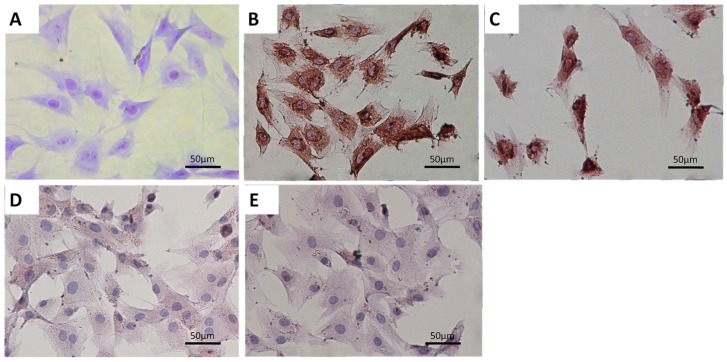
The primary mandibular condylar chondrocytes of rats were characterized using both (**A**) toluidine blue staining and (**B**–**E**) immunocytochemistry staining. These cells were positive for (**B**) type-II collagen and (**C**) aggrecan staining. They were also negative for (**D**) type I collagen and (**E**) type X collagen staining. Scale bar = 50 µm.

The primary chondrocytes showed a polygonal morphology. They displayed positive toluidine blue staining for glycosaminoglycans (Figure 1A). The primary chondrocytes were also positive for type II collagen (Figure 1B) and aggrecan staining (Figure 1C). Meanwhile, they were negative for type-I (Figure 1D) and type-X collagen staining (Figure 1E). These results demonstrated that the primary condylar chondrocytes were suitable for later experiment.

### 2.2. HCFs Could Promote the Proliferation and Decrease the Apoptosis of Condylar Chondrocytes

**Figure 2:**
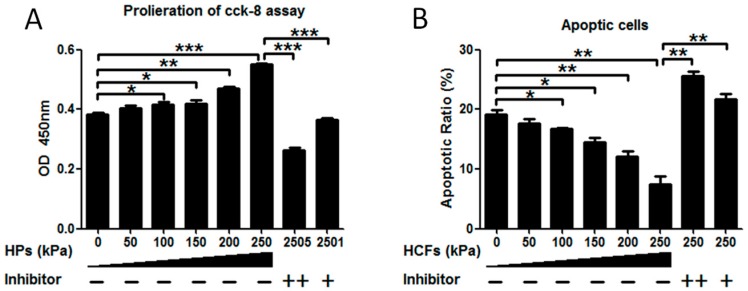
Hydrostatic compressive forces (HCFs) of different magnitudes (0–250 kPa) for 2 h significantly (**A**) enhanced the viability and (**B**) decreased the apoptosis of primary rat mandibular condylar chondrocytes in a dose-dependent manner. Such effects could completely inhibited by the integrin inhibitor—cilengitide of 1 or 5 µg/mL. All the data were presented as mean ± standard deviation (SD). *n* = 5 per group. * *p* < 0.05; ** *p* < 0.01; *** *p* < 0.001.

**Figure 3:**
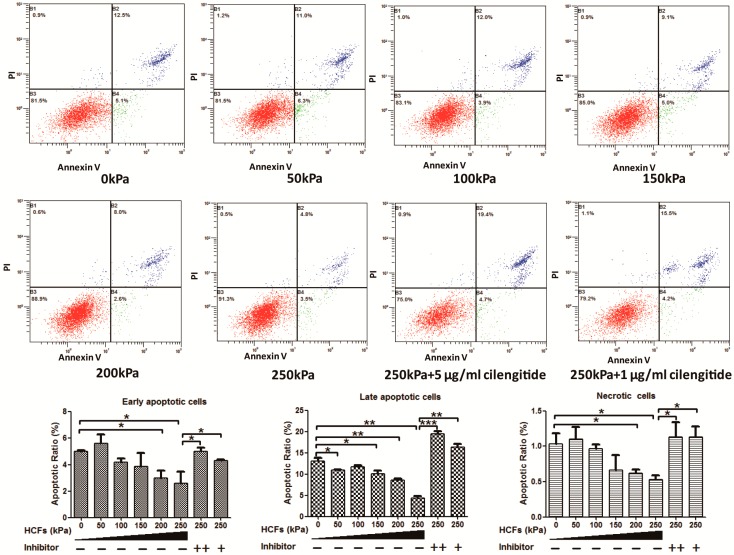
Flow cytometric analysis to detect the rates of apoptotic mandibular condylar chondrocytes under the treatment of hydrostatic compressive forces (HCFs) of different magnitudes (0–250 kPa) without or with 1 or 5 µg/mL Cilengitide. Cells were stained with FITC (Fluorescein isothiocyanate)-Annexin V and propidium iodide (PI). Viable cells were in the bottom left quadrant (red), early apoptotic cells were in the bottom right quadrant (green), the late apoptotic cells were in the upper right quadrant (blue) and the necrotic cells were in the upper left quadrant (blue). Graphs depicting the effects of HCFs on the rates of the early apoptotic, late apoptotic, and necrotic mandibular condylar chondrocytes.* *p* < 0.05; ** *p* < 0.01; *** *p* < 0.001.

When no HCFs were applied, the primary condylar chondrocytes showed a high apoptotic ratio of about 19.10% ± 0.70% (Figure 2B). HCFs of 50–250 kPa could significantly enhance the cell viability (Figure 2A) and decreased the apoptosis (Figure 2B) of condylar chondrocytes in a dose-dependent manner. HCF of 250 kPa resulted in a 1.51 ± 0.02-fold increase of cell viability and reduced the ratio of apoptotic cells from 18.10% ± 0.56% to 7.30% ± 1.43%. Flowcytometric analysis showed that 250 kPa resulted in the reduction of early apoptotic, late apoptotic, and necrotic cells by 35.36%, 61.0%, and 46.4%, respectively (Figure 3). Cilengitide, the potent integrin inhibitor, could dose-dependently block such effects of HCFs (Figure 2 and Figure 3). 

### 2.3. HCFs Enlarged the Surface Area of Condylar Chondrocytes without Changing Their Chondrocyte Phenotype

**Figure 4:**
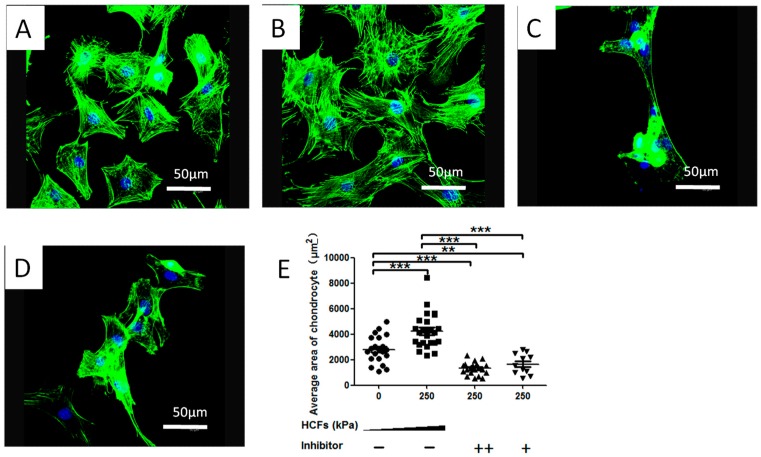
Immunofluorescent micrographs depicting the F-actin distribution in mandibular condylar chondrocytes with (**A**) no HCF treatment (0 kPa); (**B**) the treatment of 250 kPa HCF; (**C**) the treatment of 250 kPa HCF plus 1 μg/mL cilengitide; and (**D**) the treatment of 250 kPa HCF plus 5 μg/mL cilengitide; (**E**) graph depicting the surface area of cells under the abovementioned treatments. All data are presented as mean ± standard deviation (SD). ** *p* < 0.01; *** *p* < 0.001.

**Figure 5:**
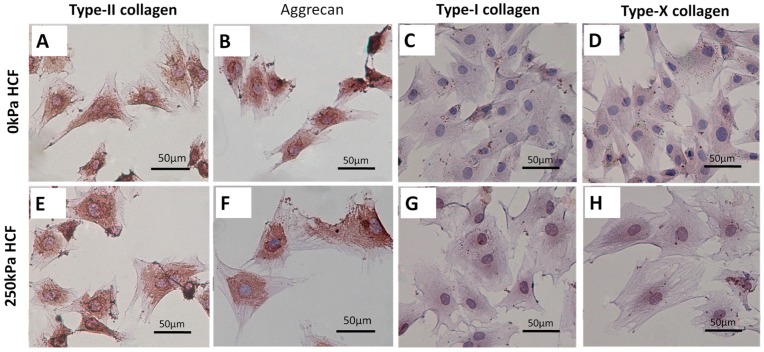
Light micrographs depicting the primary mandibular condylar chondrocytes that were treated with either 0 (**A**–**D**) or 250 kPa HCF treatment (**E**–**H**). Thereafter, these primary cells were subjected to immunocytochemistry staining for type-II collagen (**A**,**E**); aggrecan (**B**,**F**); type-I collagen (**C**,**G**); and type-X collagen (**D**,**H**). Scale bar = 50 μm.

Typical morphology alternations of condylar chondrocytes under HCFs were shown in the Figure 4. Without HCF, the condylar chondrocytes were predominantly small and polygonal (Figure 4A). In contrast, HCF of 250 kPa enhanced the cell spreading and resulted in a 1.57-fold surface area of the control group (Figure 4B,E). Such an effect of HCF was completely inhibited when treating the cells with 5 and 1 μg/mL cilengitide. After the treatment of HCFs, the condylar chondrocytes remained positive for type-II collagen (Figure 5A,E)and aggrecan (Figure 5B,F) staining, and negative for type-I collagen (Figure 5C,G) and type-X collagen (Figure 5D,H)

### 2.4. Change of Integrin α2, Integrin α5, Integrin β1, FAK, ERK1/2, and PI3K under Various HCFs

**Figure 6:**
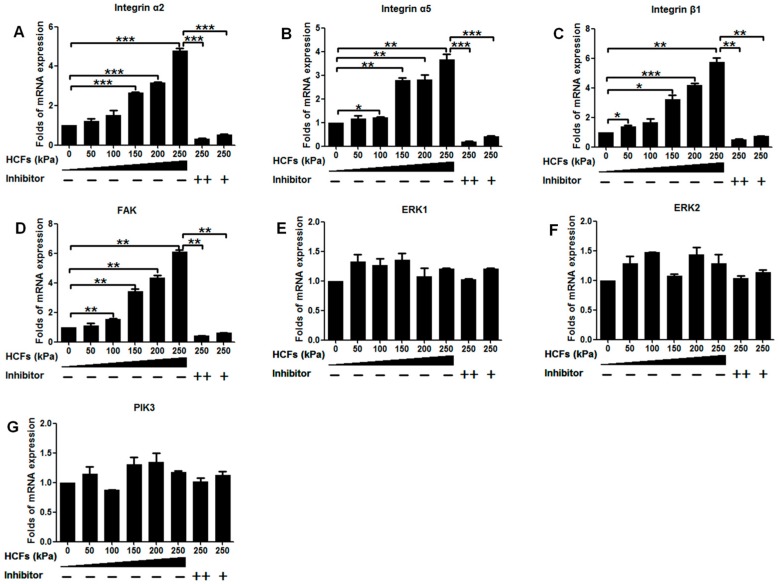
Quantitative real-time PCR analysis to detect depicting the mRNA expression levels of (**A**) integrin α2; (**B**) integrin α5; (**C**) integrin β1; (**D**) FAK; (**E**) ERK1; (**F**) ERK2; and (**G**) PI3K in mandibular condylar chondrocytes with the treatments of hydrostatic compressive forces of different magnitudes (0–250 kPa) in the presence or absence of 5 or 1 μg/mL cilengitide. All data are presented as mean ± standard deviation (SD). *n* = 5 per group. * *p* < 0.05; ** *p* < 0.01; *** *p* < 0.001.

**Figure 7:**
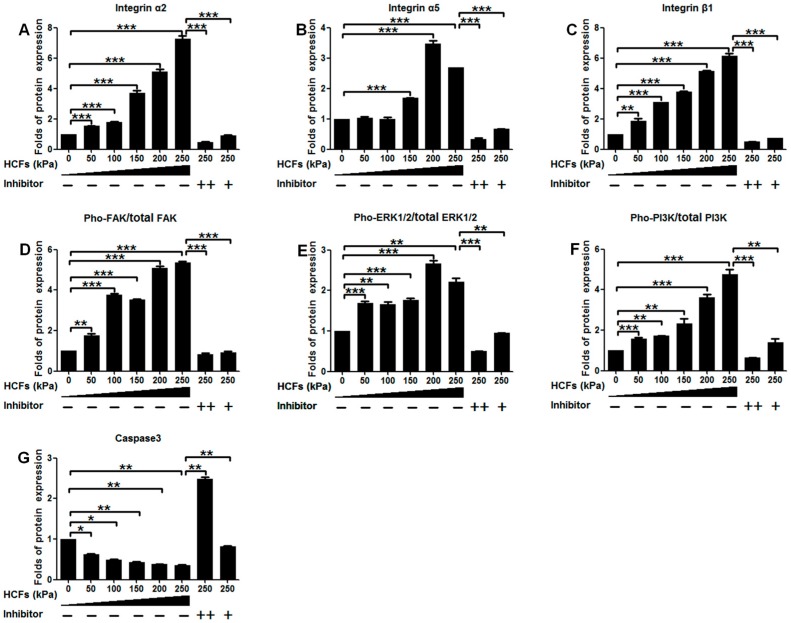

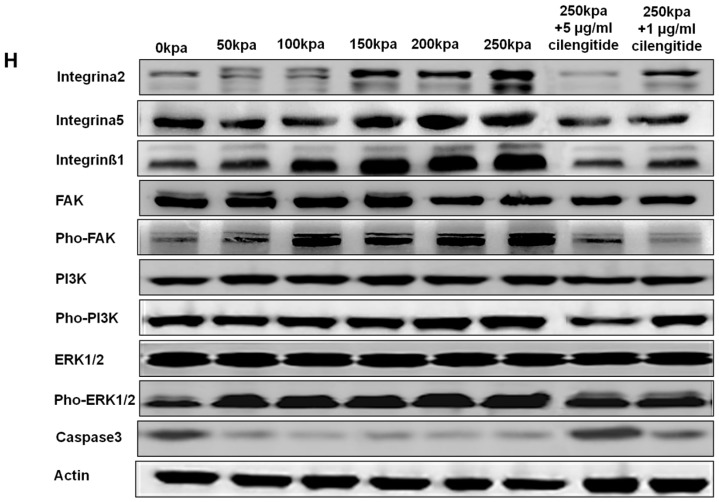
Western blotting analyses to detect the protein expression of (**A**) integrinα2; (**B**) integrin α5; (**C**) integrin β1; (**D**) Pho-FAK/FAK; (**E**) Pho-ERK/ERK, (**F**) Pho-PI3K/PI3K; and (**G**) Caspase 3 in mandibular condylar chondrocytes with the treatments of hydrostatic compressive forces of different magnitudes (0–250 kPa) in the presence or absence of 5 or 1 μg/mL cilengitide (**H**). All data are presented as mean ± standard deviation (SD). *n* = 5 per group. *n* = 5 per group. * *p* < 0.05; ** *p* < 0.01; *** *p* < 0.001.

HCFs could significantly enhance the mRNA expression of integrin α2, integrin α5, integrin β1, and FAK in a dose-dependent manner, but not ERK1, ERK2, or PI3K. HCF of 250 kPa could result in 4.8-, 3.6-, 5.8-, and 6.1-fold increases of integrin α2, integrin α5, integrin β1, and FAK mRNA expressions than the control (without HCFs), respectively (Figure 6). Consistently, HCF also dose-dependently increased the protein expression of integrin α2, integrin α5, and integrin β1 but not FAK, ERK1/2, or PI3K. Instead, HCF could significantly increase phosphorylation levels of FAK, ERK1/2, and PI3K in a dose-dependent manner (Figure 7). On the other hand, HCF could also dose-dependently decrease the protein expression of caspase-3 (Figure 7). This HCFs-induced enhancement in the mRNA, protein expression, and phosphorylation levels of the key molecules could be completely inhibited by the potent integrin inhibitor, cilengitide.

## 3. Discussion

The basic functions of the TMJ is to enable the movements of mandible for many oral functions, such as speaking, chewing, and swallowing. During these movements, mechanical forces are transmitted to condylar cartilage, which conversely play an important role in promoting the growth of condylar cartilage. A compromised mechanical stimulus caused by limited or altered masticatory functions will lead to both a decrease in growth an increase in atrophy of condylar cartilage. However, the molecular mechanisms mediating the effects of mechanical stimuli on the metabolic activities of condylar chondrocytes remain unclear. In this study, we showed that the integrin-FAK-ERK/PI3K mediated the important roles of mechanical stimuli in promoting the proliferation activities and reducing the apoptosis of primary condylar chondrocytes.

It is nearly impossible to measure the real magnitude of mechanical loadings on condylar cartilages during masticatory movements. To approach this problem, finite element models were established on the basis of magnetic resonance images from two subjects with or without anterior disc displacement of the TMJ [25,26]. The stress values after a 10-min clenching varied from 20 to 100 kPa according to the different sites of condylar cartilage [25,26]. It was also shown that rabbit chondrocytes subjected to stress ranging from 0 to 200 kPa yielded tissue-engineered cartilage of the best quality [27]. On the other hand, an unphysiologically high loading would significantly enhance the death of cartilage [28,29]. Consequently, to mimic the real mechanical stimuli and keep the comparability with previous studies, we adopted the HCFs ranging from 0 to 250 kPa in the present study. On the other hand, various types of apparatuses were used to investigate the effects of HCFs on chondrocytes. The thereby generated HCFs could be cyclic [30] or continuous (as in our study) or sinusoidal [31]. The magnitudes of HCFs could vary from from kPa to MPa and the duration also varied from hours to days. The in vitro metabolic activities of chondrocytes to HCFs varied with the magnitude, frequency, and duration of loading. Pathological microenvironments could also be created by adding pro-inflammatory cytokines to investigate the effects of HCFs on the metabolic activities of chondrocytes in osteoarhritis [32]. In addition, caution should also be taken to extrapolate the current findings to the articular cartilage-derived chondrocytes due to the unique phenotype of condylar chondrocytes [33]. As secondary cartilages (local mesenchymal cartilage formation), mandibular condylar cartilages, are fibrocartilages that are structurally distinct from both the limb growth plate and the articular cartilage. It differs from primary cartilage in patterns of the endochondral ossification, cellular organization, in vivo transplant growth, and antigenicity [34]. Under HCFs, mandibular condylar chondrocytes reacted differentially both in time-dependent profile and magnitudes in comparison with primary cartilage-derived chondrocytes [34]. 

When the primary condylar chondrocytes were isolated and cultured on cover slides in vitro, they showed a very high rate (11%–30%) of apoptosis [35]. Consistent with this result, the primary condylar chondrocytes cultured on cover slides exhibited an apoptotic ratio of 18.10% ± 0.56% (Figure 3). Such apoptosis might be due to the lack of stimulating signals for maintaining the survival of primary chondrocytes in this unphysiological and suboptimal microenvironment [35]. It was previously shown that cell-matrix interactions via integrins were necessary for all chondrocytes to survive [19,35]. The metabolic activity of primary chondrocytes was also associated with mechanical signals, whose effects were also mediated by integrins [27]. Furthermore, we also showed that HCF could not only significantly enhance the viability (Figure 2A) but also decrease apoptosis of primary chondrocytes in a dose-dependent manner (Figure 2B). Consistently, we also showed that expression of caspase-3 could be significantly reduced by HCFs (Figure 7G). Furthermore, our data showed that HCF could significantly and dose-dependently promote the expression of integrin β1, integrin α2, and integrin α5 both in transcriptional levels (mRNA expression) and translational levels (protein expression) in the primary condylar chondrocytes (Figure 6 and Figure 7). Such effects could be completely inhibited by the competitive inhibitor of integrins (Figure 6 and Figure 7). In fact, integrins, such as integrin β1 and integrin α5 are the major mediators of mechanical signals in chondrocytes [36] but also in many other cell types, such as in myocardial cells [37] and fibroblasts [38]. Consequently, integrins are very likely to be the converging molecular harbor for sensing the signals from cell matrix and mechanical forces, thereby playing an important role in modulating the viability and apoptosis of condylar chondrocytes.

FAK, a cytoplasmic tyrosine kinase located in the focal adhesion complex, has been implicated in the signal transduction from integrins [39,40]. FAK played an anti-apoptotic role [24,41,42] FAK acted as an upstream regulator of the ERK1/2 signaling upon mechanical stimulation in many non-chondrocytic cell types [21,22]. The downregulation of FAK could significantly reduce ERK and Akt survival signaling, subsequently leading to apoptosis [43]. This association of FAK-Src, together with the Grb2 adaptor protein, represents one mechanism for the activation of ERK by FAK [44]. The pho-ERK1/2 could migrate from the cytosol to the nucleus where they regulate the activity of transcription factors [45]. The importance of pho-ERK1/2 to chondrocyte survival was corroborated by the fact the specific inhibition of ERK1/2 activation could result in apoptosis of human chondrocytes [46]. FAK could also directly activate PI3K [47,48]. Consistent with those previous reports, our data showed that Pho-ERK1/2 and Pho-PI3K were significantly promoted in a dose-dependent manner in response to HCFs (Figure 7). Furthermore, the inhibition of integrins could completely inhibit the elevation of Pho-ERK1/2 and Pho-PI3K. These data indicated that ERK and PI3K were actively involved in the mechanical forces-induced integrin signaling transduction. 

Hitherto, it remains not clarified how mechanical forces modulate caspase in chondrocytes. Previous studies suggested that the inhibited integrin-ligand interactions by Arg–Gly–Asp peptides could cause apoptosis through the conformational changes in the uncleaved caspase-3 molecule, leading to its auto-cleavage and generation of active caspase-3 molecules in anchorage-dependent cells [49]. Consequently, for the isolated primary chondrocytes, the reduced interactions of integration with cellular matrix might cause the increased expression of caspase-3, thus resulting in a very high apoptotic rate (Figure 2B). Consistent with this principle, the activation of integrin was rescued by HCFs in our study (Figure 6 and Figure 7), which could directly reduce caspase-3 (Figure 7G) and apoptosis (Figure 2 and Figure 3). On the other hand, FAK could also regulate the activity of caspase by facilitating the marked induction of inhibitor-of-apoptosis proteins, by a proposed mechanism involving PI3K/Akt activation of the NF-κB pathway [24]. Further studies must be performed to identify the mechanism accounting for the modulation of caspase by mechanical forces in chondrocytes.

HCFs could significantly enhance the surface area of the primary condylar chondrocytes. HCF of 250 kPa resulted in a 1.57-fold surface area of the control group (Figure 4). Meanwhile, the cell viability and proliferation index of the chondrocytes were significantly enhanced (Figure 2). It was previously suggested that this shape change has been associated with a number of biochemical changes. In mammalian cells, a high correlation has been reported between cellular shape and proliferation [50,51,52]. A greater mitotic activity was observed among the flattest cells. In contrast, minimal DNA synthesis was found among the roundest cells. In addition, we found that the condylar chondrocytes remained positive for aggrecan and type-II collagen staining, and negative for type-I and type-X collagen staining (Figure 5). These findings suggested that the HCFs affected the metabolic activities of the condylar chondrocytes without significantly changing their chondrocytic phenotype. Indeed, flat polygonal vertebral chondrocytes in culture showed a progressive increase in size and type-X collagen gene expression as a function of time in culture [53]. Similar changes were found during the maturation processes of epiphyseal cartilages [54]. Our experiments confirmed these observations, since the greatest increase in cell number occurred under various magnitude of HCF in culture, just after the cells had extensively spread out. Meanwhile, a marked decrease in cell viability occurred to the cells that shrank when treated with the integrin inhibitor, cilengitide. The results suggested that integrins also mediated the HCFs-induced morphological changes.

One of the limitations in this study is the adoption of a monolayer culture system instead of a 3D culture system. A 3D culture system may more simulate the real microenvironments of condylar chondrocytes, such as three-dimensional anchorage and signals from cell matrix [55,56,57], which may significantly affect the mechanosensitivity of condylar chondrocytes. In our future study, we will develop a 3D culture system and access the cellular response of condylar chondrocytes to HCFs. The pattern of HCFs could also be modulated, such as cyclic pattern or with different treatment durations. Further, more specific inhibitors could be adopted to illustrate the influences of FAK, ERK, and PI3K.

## 4. Materials and Methods

### 4.1. Isolation and Culture of Primary Mandibular Condylar Chondrocytes

Mandibular condylar chondrocytes were harvested and cultured as previously reported [58]. Briefly, mandibular condylar cartilage was isolated from Sprague–Dawley (SD) neonatal rats less than three weeks of age and was subsequently minced. After digestion with 0.25% trypsinase and 0.2% collagenase, primary condylar chondrocytes were rinsed three times and prepared as a single cell suspension in a growth medium of DMEM (Dulbecco’s Modified Eagle’s medium) supplemented with 10% FCS (fetal calf serum), 25 mM HEPES, 1% l-glutamine, and 100 mg/mL kanamycin. Cells were seeded and incubated overnight at a high density of 1 × 10^6^ cells/cm^2^ in a humidified atmosphere at 37.8 °C and 5% CO_2_. The third-generation condylar chondrocytes were validated by toluidine blue staining a series of immunohistochemistry staining. The cultured chondrocytes were stained with toluidine blue as previously reported [59]. For immunochemistry staining, mandibular condylar chondrocytes were fixed in 4% formalin solution for 15 min and permeabilized with 0.3% Triton X-100 in phosphate-buffered saline (PBS) for 10 min. The fixed cells were then incubated with the primary antibodies, such as anti-type-II collagen (ab34712, Abcam, Cambridge, UK, 1:100), anti-aggrecan (ab36861, Abcam, UK, 1:100), anti-type-X collagen (ab58632, Abcam, UK, 1:500), and anti-type-I collagen (ab34710, Abcam, UK, 1:500) antibodies. The Vectastain Elite ABC Peroxidase kit (Vector Laboratories, Inc., Burlingame, CA, USA) was used for secondary antibody detection. Visualization was done by using DAB as a substrate (ab64238, Abcam, UK). The images were acquired by light microscopy. 

### 4.2. Application of Hydrostatic Compressive Forces and Integrin Inhibitor

**Figure 8:**
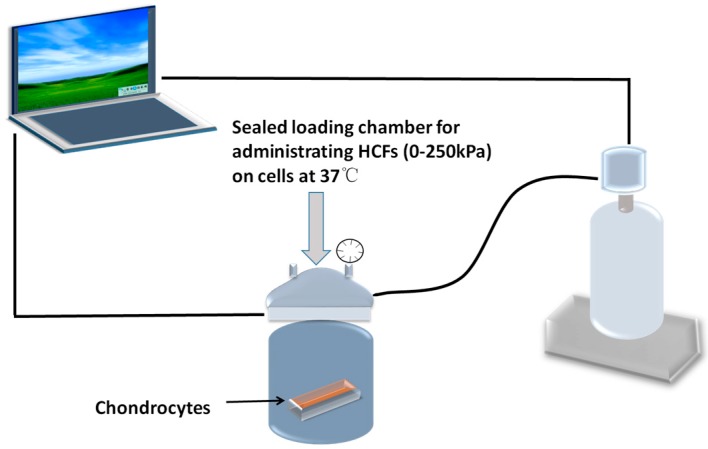
Schematic graph depicting the apparatus for administrating HCFs on mandibular condylar chondrocytes.

The HCF loading system was designed and used as we previously reported [60]. HCFs were applied to monolayer of condylar chondrocytes by an in-house designed computer-controlled pressure chamber (Figure 8). The apparatus was manufactured by SASPG Medical Equipment Factory (Chengdu, China). The apparatus used in this study was designed according to description of Smith and Yamamoto et al. [61,62] with a minor modification. This apparatus enabled the administration of HCFs of 0–250 kPa with a temperature control at 37 °C under sterile condition. The gas phase of the chamber was maintained at a pressure of scheduled magnitude by continuously infusing a compressed mixed gas (O_2_:N_2_:CO_2_ = 7.0%:91.3%:1.7%) [63]. Our preliminary experiments have confirmed that the pH of the DMEM was constant at 7.4 and the temperature was maintained at 37 °C before and after the experiment [60]. Cells that were seeded in tissue culture dish and placed in the same apparatus under the same conditions were used as controls (0 kPa). All specimens were harvested after the HCFs treatment for 2 h. Chondrocytes (1 × 10^6^ cells/mL, 0.2 mL) were grown in 96-well plates and incubated for 2 h with HCFs of different magnitudes (0, 50, 100, 150, 200, and 250 kPa). To block the integrin-dependent signaling, 1 or 5 µg/mL cilengitide, a specific integrin inhibitor [64], was applied 24 h before the application of 250 kPa HCF.

### 4.3. Cell Viability Assay

The viability of the cells after HCF treatment was determined over a seven-day period using CCK-8 solution according to the manufacturer’s instructions [63]. Cell were added to 10 µL of CCK-8 solution in each well of five 96-well plates (*n* = 5) and incubated for 4 h at 37 °C. The absorbance of each well was determined at 450 nm using a microplate [65]. 

### 4.4. Detection of Cell Apoptosis

Mandibular condylar chondrocytes were incubated on uncoated dishes with and without cilengitide (1 and 5 μg/mL) for 24 h at 37 °C. Cells were double-stained by using an Annexin V-FITC apoptosis detection kit (ab14085, Abcam, Cambridge, UK). Annexin staining was performed according to the manufacturer’s instructions. In brief, condylar chondrocytes were collected by centrifugation, re-suspended cells in 500 μL of 1× Binding Buffer and incubated with 5 μL of annexin V-FITC and 5 μL of propidium iodide (PI 50 µg/mL, optional) for 5 min in the dark at a room temperature. Mandibular condylar chondrocytes were quantitatively analyzed at 488 nm emission and 530 nm excitation by Flow Cytometry (BD FACScalibu; BD Biosciences, San Jose, CA, USA). FACS (rluorescence-activated cell sorting) analyses were performed as described previously [66]. Apoptosis was assessed after the staining with FITC-labeled Annexin-V and PI (BD Pharmingen, San Jose, CA, USA). Positive staining with FITC-labeled Annexin-V reflects a shift of phosphatidylserine from the inner to the outer layer of the cytoplasmatic membrane, which occurs early in apoptosis. Annexin-V-positive and PI-negative cells were scored as early apoptotic cells. Cells labeled by Annexin V and PI have been determined as late apoptotic. Annexin-negative and PI-positive events display necrotic cells.

### 4.5. Actin Staining and Estimation of Cell Surface Area

Condylar chondrocytes seeded on slide glass with density of 1 × 10^6^ cells/cm^2^ were treated with different HCFs according to the protocol mentioned above. Cells were rinsed in PBS, fixed in 4% paraformaldehyde in neutralized PBS for 30 min, permeabilized with 0.5% Triton X-100 (Sigma, St. Louis, MO, USA), blocked with 6% fetal bovine serum (Shanghai Sangon Biological Engineering Technology & Services Co., Shanghai, China), and incubated with 2 U/mL rhodamine-phalloidin (Sigma) for 40 min, 5 mg/mL propidium iodide (Sigma) for 10 min. Fluorescence was examined with a fluorescent microscope (Olympus CX-RFL-2, Tokyo, Japan). The surface area of each cell was estimated using a point-counting method [67].

### 4.6. Quantitative RT**-**PCR

Total RNA was extracted using Trizol reagent (Invitrogen, Carlsbad, CA, USA) and was reverse-transcribed to cDNA using a Reverse Transcription System (Promega, Madison, WI, USA). The input cDNA was standardized and then amplified for 45 cycles with SYBR Green Master Mix and gene-specific primers on an ABI Prism 7900HT machine (Applied Biosystems, Foster City, CA, USA); endogenous GAPDH was regarded as an internal control, and samples were analyzed in triplicate. Primers and probes were designed using annotated sequences from the porcine database and Applied Biosystem Primer Express software. The sequences of synthesized primers and probes are shown in Table 1. Each well was loaded with 2.5 µL SYBR green master mix, 1.2 µL H_2_O, and 0.15 µL of each primer (10 µM; final concentration, 250 nM). 1 µL cDNA was amplified using a standardized program with a 10 min denaturing step following 45 cycles of 5 s 95 °C, 15 s 65 °C, and 15 s at 72 °C, melting point analysis in 0.1 °C steps, and a final cooling step. Relative quantification of target gene expression was calculated according to SDS2.4 RQ manager (Applied Biosystems, Foster City, CA, USA). The PCR efficiencies for target gene (amplicons) were calculated using the Relative Quantification Software from ABI Molecular Biochemicals (Applied Biosystems, Foster City, CA, USA). Target gene mRNA was normalized according to the relative content of the target gene under each treatment condition. Results were shown as fold inductions of target gene mRNA compared with the untreated control.

### 4.7. Western Blot Analysis

Total cellular proteins were extracted using Mammalian protein extraction reagent (Sigma-Aldrich, St. Louis, MO, USA) with 1× Roche cocktail protease inhibitor mixture. The lysates were collected, centrifuged at 12,000 rpm using a microcentrifuge (Eppendorf, Germany) for 10 min at 4 °C, pellets were discarded, and supernatants were transferred to a new Eppendorf tube. Protein was quantified using the BCA protein assay kit (Pierce BCA protein assay kit, Thermo Fisher scientific, Waltham, MA, USA). Samples were boiled in an equal volume of SDS loading buffer for 10 min and separated on 10% to 12% SDS-PAGE gels. Proteins resolved on the gels were transferred to 0.22 μm polyvinylidenedifluoride (PVDF) membranes (Millipore, Billerica, MA, USA) for 2 h at 90 V, according to standard procedures. The bolts were incubated and nonspecific binding sites were blocked with 5% BSA/TBS-Tween 20 followed by incubation in primary antibodies at 4 °C overnight and then washed three times in the 1× TBS-Tween 20 buffer. Horseradish Peroxidase (HRP)-conjugated anti-mouse or anti-rabbit secondary antibodies were used at 1:750 (Santa Cruz Biotechnology, Santa Cruz, CA, USA). Detection was performed using Super Signal West Femto Chemiluminscence Substrate (Thermo Fisher scientific, Waltham, MA, USA). The blots were visualized using an Image Quant LAS4000 biomolecular imager (GE Healthcare, Bethesda, MD, USA). Proteins were normalized to the level of β-actin protein.

### 4.8. Statistical Analysis

Data were expressed as mean ± standard deviation (SD). Statistical analysis was performed using unpaired *t*-test or one-way analysis of variance depending on whether the data were normally distributed. All statistical analysis was done in SPSS version 20.0 (IBM, Armonk, NY, USA), *p* < 0.05 was considered to be statistically significant.

## Figures and Tables

**Table 1:** Primer sequences for real-time quantitative polymerase chain reaction analysis of the mRNA expression of *Itga2*, *Itga5*, *Itgb1*, *FAK*, *ERK1*, *ERK2*, *PI3K1*, and *GAPDH* genes.

Gene	Accession No.	Primers (5′-3′) (F = Forward; R = Reverse)
*Itga2*	XM_001075558.5	F: TGGAATTTGTTCTGATGTCAGTCC
		R: GTGGTCAAGTTAAAGACAACTCTT
*Itga5*	NM_001314041.1	F: TGCAGCACCATTCAATTTGACAGC
		R: TCATTCTGTGGGTCCTTTTCTGTG
*Itgb1*	XM_006255824.1	F: AATTCAAGAGGGCTGAAGACTAC
		R: TGTCAGTAAGACTAAGCACG
*FAK*	XM_006520433.2	F: CCAAGTTCGAGTACTAAGACTCACC
		R: AAATCCATAGCAGGCCACGTGC
*ERK1*	NM_017347.2	F: AGAGATCATGCTTAACTCCAAG
		R: TTCATGTTAATGATACAATTTAGGTCCTC
*ERK2*	XM_006522147.3	F: CTGCACCGTGACCTCAAGCC
		R: CAATGGACTTGGTATAACCCTTGG
*PI3K1*	XM_006240005.2	F: CTGATTGGCTACGACGTCAC
		R: GAAGAAGCTCTGAAGGATGGTGTC
*GAPDH*	XM_017321385.1	F: AAAGGCATCTTGGGCTACACCG
		R: ATGAGGTCCACCACCCTGTTG
